# World Tuberculosis Day 2021: keeping an eye on the 2030 target

**DOI:** 10.2807/1560-7917.ES.2021.26.11.2103181

**Published:** 2021-03-18

**Authors:** Eurosurveillance editorial team

**Affiliations:** 1 European Centre for Disease Prevention and Control (ECDC), Stockholm, Sweden

**Keywords:** tuberculosis, TB, epidemic

The United Nations Sustainable Development Goal (SDG) 3 sets the target of ending the global tuberculosis (TB) epidemic by 2030 [1]. The End TB Strategy [2], developed by the World Health Organization (WHO), outlines the sub-targets used to measure progress towards the SDG. In addition, political commitments have been made to achieve the targets [3].

At present, however, the coronavirus disease pandemic remains the main focus of public health officials and policymakers. The pandemic is dominating the news and day-to-day life, while also impacting the prevention and control of other diseases, worldwide. World TB Day, observed annually on 24 March, provides an opportunity for the public health community to shift focus and raise awareness of the burden of TB and the current status of TB prevention and control efforts. The theme of World TB Day 2021, ***‘*** *The clock is ticking’*, reminds us that we must continue efforts to progress towards the agreed-upon TB targets if we are to end TB by 2030.

Childhood TB cases can be understood as a sign of ongoing TB transmission within a community, as TB disease in children indicates recent infection [4]. Of the 50,455 newly reported TB cases and relapses across European Union/European Economic Area (EU/EEA) countries in 2018, children under 15 years of age accounted for 2,035 cases (4%) and had a lower notification rate than adult age groups [5].

Vaccination with the *Bacillus* Calmette-Guérin (BCG) vaccine protects infants and children against severe forms of TB. Universal BCG vaccination among infants is still common in the EU/EEA, but a number of countries with declining TB incidence have moved towards vaccination of selective groups only [6]. However, there is as yet limited evidence to determine the threshold incidence for discontinuing universal BCG vaccination [7].

Marking World TB Day 2021, this issue of *Eurosurveillance* includes the article ‘Paediatric tuberculosis during universal and selective *Bacillus* Calmette–Guérin vaccination policy: a nationwide population-based retrospective study, Finland, 1995–2015’ by Kontturi et al. The article reports on Finland’s policy change from universal BCG vaccination to selective BCG vaccination that is only for children identified as having higher risk of TB exposure. The authors conclude that in settings with low TB incidence, selective BCG vaccination can prevent TB among the most vulnerable almost as effectively as universal vaccination [8].

Also on the occasion of World TB Day, the journal has compiled a new collection of TB articles published between 2019 and 2021, which is available on our website.

## References

[1] United Nations. Goals: 3 Ensure healthy lives and promote well-being for all at all ages. Targets and Indicators. New York: United Nations. [Accessed: 16 Mar 2021]. Available from: https://sdgs.un.org/goals/goal3

[2] World Health Organization (WHO). Tuberculosis (TB). The End TB Strategy. Geneva: WHO; 2018. Available from: https://www.who.int/tb/strategy/end-tb/en/

[3] United Nations. Resolution adopted by the General Assembly on 10 October 2018 [without reference to a Main Committee (A/73/L.4)] 73/3. Political declaration of the high-level meeting of the General Assembly on the fight against tuberculosis. Official Journal of the United Nations. New York: United Nations. 18.10.2018:A/RES/73/3. Available from: https://www.un.org/en/ga/search/view_doc.asp?symbol=A/RES/73/3

[4] European Centre for Disease Prevention and Control (ECDC). Investigation and control of tuberculosis incidents affecting children in congregate settings. Stockholm: ECDC; 2013. Available from: https://www.ecdc.europa.eu/en/publications-data/public-health-guidance-investigation-and-control-tuberculosis-incidents-affecting

[5] European Centre for Disease Prevention and Control (ECDC), World Health Organization Regional Office for Europe. (WHO/Europe). Tuberculosis surveillance and monitoring in Europe 2020 – 2018 data. Stockholm, Copenhagen: ECDC, WHO/Europe; 2020. Available from: https://www.ecdc.europa.eu/en/publications-data/tuberculosis-surveillance-and-monitoring-europe-2020-2018-data

[6] European Centre for Disease Prevention and Control (ECDC). Vaccine Scheduler. Stockholm: ECDC. [Accessed: 16 Mar 2021]. Available from: https://vaccine-schedule.ecdc.europa.eu/Scheduler/ByDisease?SelectedDiseaseId=14&SelectedCountryIdByDisease=-1

[7] Faust L Schreiber Y Bocking N. A systematic review of BCG vaccination policies among high-risk groups in low TB-burden countries: implications for vaccination strategy in Canadian indigenous communities. BMC Public Health. 2019;19(1):1504. 10.1186/s12889-019-7868-9 31711446 PMC6849173

[8] Kontturi A Kekomäki S Soini H Ollgren J Salo E. Paediatric tuberculosis during universal and selective Bacillus Calmette–Guérin vaccination policy: a nationwide population-based retrospective study, Finland, 1995–2015. Euro Surveill. 2021;26(11):1900711. 10.2807/1560-7917.ES.2021.26.11.1900711 PMC7976386 33739257

